# Cost-effectiveness of treating relapsed or refractory 3L+ follicular lymphoma with axicabtagene ciloleucel vs mosunetuzumab in the United States

**DOI:** 10.3389/fimmu.2024.1393939

**Published:** 2024-05-24

**Authors:** Olalekan O. Oluwole, Markqayne D. Ray, Richard M. Zur, Cheryl P. Ferrufino, Brett Doble, Anik R. Patel, S. Pinar Bilir

**Affiliations:** ^1^ Vanderbilt University Medical Center, Nashville, TN, United States; ^2^ Kite, A Gilead Company, Santa Monica, CA, United States; ^3^ IQVIA, Falls Church, VA, United States

**Keywords:** axicabtagene ciloleucel, mosunetuzumab, cost-effectiveness analysis, relapsed or refractory follicular lymphoma, indolent non-Hodgkin lymphoma

## Abstract

**Introduction:**

Novel therapies for 3L+ relapsed/refractory (r/r) follicular lymphoma (FL) have been approved recently by the US Food and Drug Administration including anti-CD19 CAR-T therapies such as axicabtagene ciloleucel (axi-cel) and CD20 × CD3 T-cell-engaging bispecific monoclonal antibodies such as mosunetuzumab (mosun). The objective of this study was to assess the cost-effectiveness of axi-cel compared to mosun in 3L+ r/r FL patients from a US third-party payer perspective.

**Methods:**

A three-state (progression-free, progressed disease, and death) partitioned-survival model was used to compare two treatments over a lifetime horizon in a hypothetical cohort of US adults (age ≥18) receiving 3L+ treatment for r/r FL. ZUMA-5 and GO29781 trial data were used to inform progression-free survival (PFS) and overall survival (OS). Mosun survival was modeled via hazard ratios (HRs) applied to axi-cel survival curves. The PFS HR value was estimated via a matching-adjusted indirect comparison (MAIC) based on mosun pseudo-individual patient data and adjusted axi-cel data to account for trial populations differences. One-way sensitivity analysis (OWSA) and probabilistic sensitivity analyses (PSA) were conducted. Scenario analyses included: 1) the mosun HRs were applied to the weighted (adjusted) ZUMA-5 24-month data to most exactly reflect the MAIC, 2) mosun HR values were applied to axi-cel 48-month follow-up data, and 3) recent axi-cel health state utility values in diffuse large B-cell lymphoma patients.

**Results:**

The analysis estimated increases of 1.82 LY and 1.89 QALY for axi-cel compared to mosun. PFS for axi-cel patients was 6.42 LY vs. 1.60 LY for mosun. Increase of $257,113 in the progression-free state was driven by one-time axi-cel treatment costs. Total incremental costs for axi-cel were $204,377, resulting in an ICER of $108,307/QALY gained. The OWSA led to ICERs ranging from $240,255 to $75,624, with all but two parameters falling below $150,000/QALY. In the PSA, axi-cel had an 64% probability of being cost-effective across 5,000 iterations using a $150,000 willingness-to-pay threshold. Scenarios one and two resulted in ICERs of $105,353 and $102,695, respectively.

**Discussion:**

This study finds that axi-cel is cost-effective compared to mosun at the commonly cited $150,000/QALY US willingness-to-pay threshold, with robust results across a range of sensitivity analyses accounting for parameter uncertainty.

## 1 Introduction

Indolent non-Hodgkin lymphoma (iNHL) is a type of slow-growing lymphoma that accounts for approximately one-third to 40% of NHL cases (1), with follicular lymphoma (FL) being the most common subtype, comprising about 20% of all NHL cases in the United States (US) (2). The prevalence of NHL has increased in recent decades due to improved survival and aging of the population (3). At the same time, mortality of NHL has also decreased since the late 1990s due to the introduction of new treatments such as monoclonal antibodies and radioimmunotherapy, and more recently chimeric antigen receptor T-cell (CAR-T) therapy and bispecific antibodies. The median age at diagnosis of FL is 65 years (4), with median overall survival (OS) of 25 years and asymptomatic presentation in early stages (5). However, people diagnosed with Stage IV FL have a markedly reduced median survival time of 8.7 years. While OS after first-line therapy is as high as 25 years, it decreases with each additional line of therapy; median OS for 3^rd^-line therapy is 8.8 years, while for 6^th^-line therapy, median OS is 1.9 years (6). Given that patients will experience toxicities from multiple sequential lines of therapy and eventually develop treatment refractory disease, optimal treatment continues to be an unmet need (7, 8).

Novel therapies for 3L+ relapsed/refractory (r/r) follicular lymphoma (FL) have been approved recently by the US Food and Drug Administration including anti-CD19 CAR-T therapies (9), such as axicabtagene ciloleucel (axi-cel, manufactured by Kite Pharma), which is a type of immunotherapy that uses genetically modified T cells to target and kill cancer cells (10), and CD20 × CD3 T-cell-engaging bispecific monoclonal antibodies, such as mosunetuzumab (mosun, manufactured by Genentech, Inc.) which redirects T cells to attack malignant B cells (11).

Given the recent development of both treatments, decision-makers including providers, patients, and payers may have difficulty understanding the relative value of mosun and axi-cel. For instance, no head-to-head evaluation between axi-cel and mosun is available yet. However, there are some indications of comparative efficacy. Matching-adjusted indirect comparison (MAIC) uses propensity score weighting to adjust for differences across trial populations, and two recently-published MAICs showed longer progression-free survival (PFS) for patients receiving axi-cel rather than mosun in the third line (12, 13). Median overall survival (OS) had not yet been reached for either treatment at the time of analysis (14, 15) making it impossible to create a comparative analysis on that endpoint. Beyond efficacy, decision-makers may also find that quality of life plays a role in their treatment choice, which could be driven by differences in the treatments’ safety profiles as well as their infusion schedules (5, 6, 16, 17).

Translating these efficacy, safety, and quality of life profiles into longer-term economic outcomes may also be an important consideration in understanding the relative value of each therapy over time. Cost-effectiveness analysis is a method for such an evaluation, formally assessing clinical and cost outcomes between potential choices (18). This type of analysis leads to an incremental cost-effectiveness ratio (ICER) that quantifies incremental costs per incremental health benefit (in many cases, the quality-adjusted life year, or QALY). Although the use of the ICER is more formalized in approval decisions for coverage of new treatments outside the US, there is increasing interest and use in recent years in the US, with a general understanding that ICER values under $150,000/QALY are considered acceptable (19–21). To this end, modeling the associated economic consequences of treatment options can add to the body of evidence that guides treatment decisions in r/r FL.

Therefore, the objective of this study was to model lifetime economic outcomes and thus assess the cost-effectiveness of axi-cel compared to mosun in r/r FL patients who have had at least two lines of prior therapy from a US third-party payer perspective.

## 2 Materials and methods

### 2.1 Cost-effectiveness model structure

**Figure 1:**
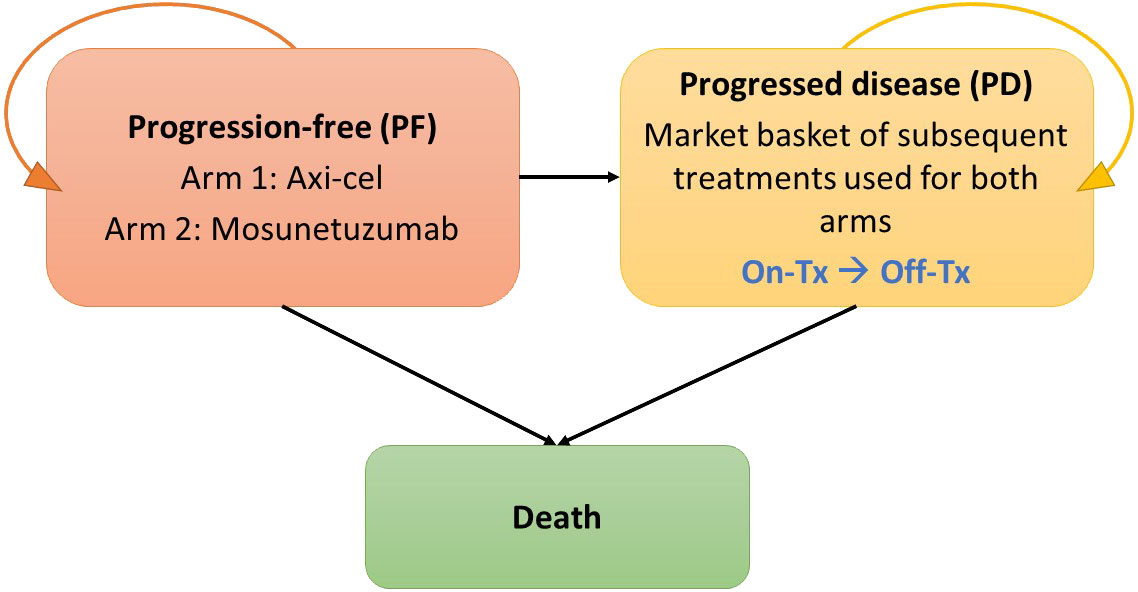
Illustration of Partitioned Survival Model.

A cost-effectiveness model was built in MS Excel 365, based on a partitioned-survival calculation structure with progression-free (PF), progressed disease (PD; with sub-states for on- and off-treatment), and death health states, and is illustrated in **Figure 1**. This cost-effectiveness analysis models the cost and outcomes, such as PF survival, overall survival and survival adjusted for quality of life, of a simulated cohort of r/r FL patients responding to different treatment scenarios. Using standard cost-effectiveness analytic methods, the differences between the two treatment scenarios are reported as incremental costs and incremental effects, with effects typically reported as life years of survival or quality-adjusted life years (22). The modeled patient population reflected US adults aged 18 and older with r/r FL who have had at least two lines of prior systemic therapy. All patients started in the PF state and transitioned either to PD or death based on parametric PFS curves or overall survival (OS) curves. A MAIC analysis based on mosun pseudo-individual patient data from GO29781 (NCT02500407) (17) and adjusted axi-cel ZUMA-5 (NCT03105336) 24-month follow-up data (16) was used to estimate a hazard ratio (HR) (13). In the base case, mosun survival curves were estimated using the MAIC HR applied to axi-cel survival curves estimated using full ZUMA-5 data. Full data were used given the large overlap in key prognostic factors in trial population. Scenario analyses (Section 2.5) explore alternative ZUMA-5 data. Patients in the PF state could remain PF, progress or die but were never permitted to return to the PF state. PD was divided into on- and off-treatment to capture the different health utilities associated with or without treatment, and patients could remain in this state or die.

After failure of the initial 3L+ therapy, patients initiated subsequent lines of treatment. The model assumed that subsequent lines of treatment had no differential impact on survival between comparators and that modeled survival differences were driven by the primary 3L+ survival curves.

The base case analysis used a lifetime horizon (i.e., all patients progressed to death), and a 3% discount rate was applied to costs (2023 USD) and clinical outcomes (life years (LYs), QALYs) according to US modeling guidelines (23). Reporting was guided by the CHEERS checklist (24).

### 2.2 Application of clinical data to drive model structure

ZUMA-5 is a multicenter, single-arm Phase 2 study of axi-cel patients with r/r iNHL (FL or MZL) who have been treated with two or more lines of therapy (25). The axi-cel survival analysis was performed on 24-month ZUMA-5 FL patient-level data. Although longer-term ZUMA-5 data are available, an independent review committee assessment of outcomes determined that the 24-month data would most closely align with available mosun trial data for purposes of MAIC analysis (13). While 24-month data thus underpin the base case analysis, scenario analysis is performed with the recently published 48-month ZUMA-5 follow-up data to explore the impact on results, assuming that the mosun PFS HR would remain the same.

Parametric models were fit to OS and PFS Kaplan Meier (KM) data, as these reflect the full trial population, to extrapolate outcomes over a lifetime time horizon. The following parametric curves were tested: exponential, Weibull, Gompertz, log-logistic, generalized gamma, gamma, and log-normal. Model fit was assessed via minimizing the Akaike information criterion and Bayesian information criterion as well as visual inspection and clinical review for plausibility of long-term extrapolation. Exponential models were considered most appropriate for both the OS and PFS curves.

**Figure 2:**
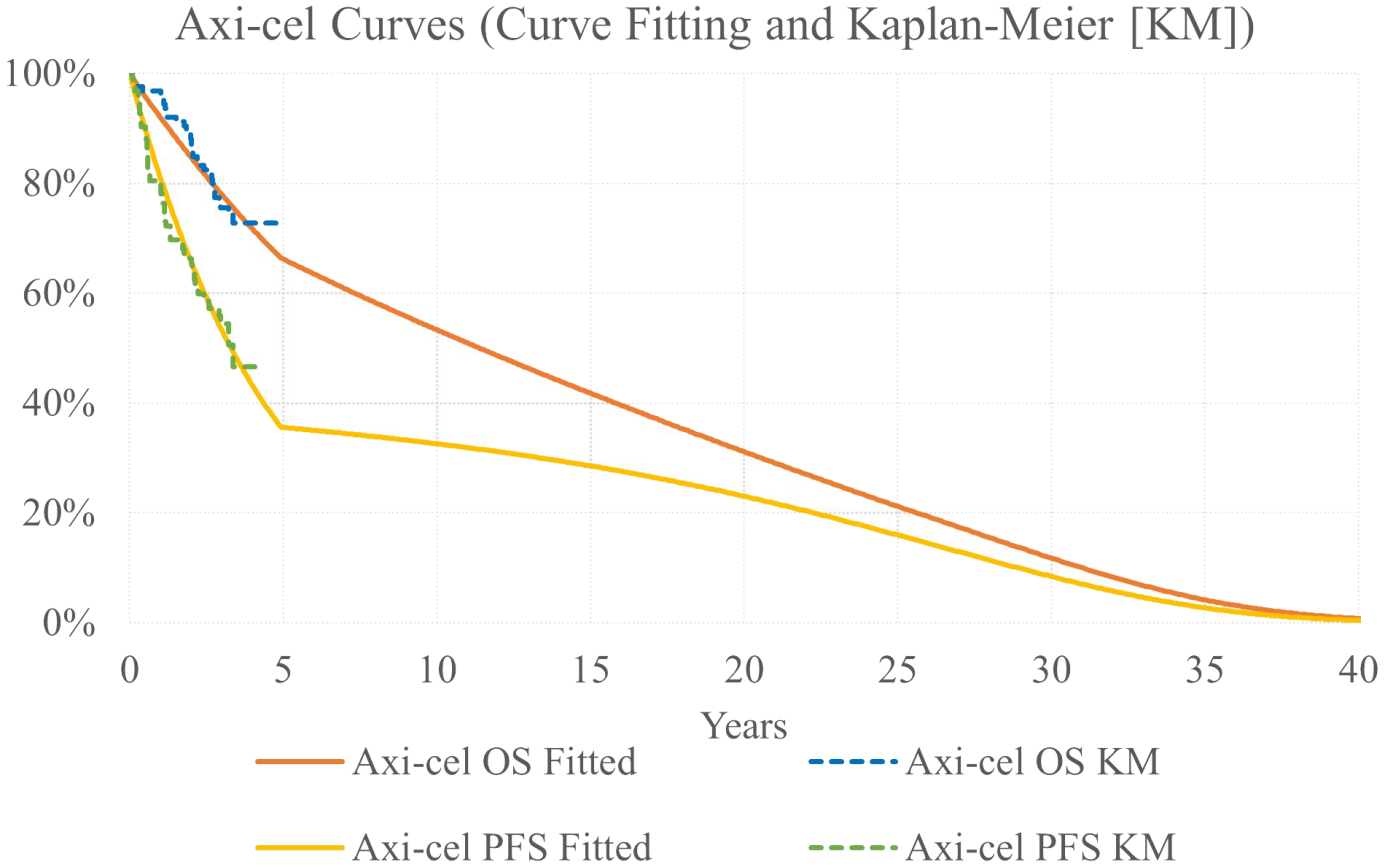
Axi-cel OS and PFS Kaplan-Meier Data [KM] and Parametric Survival Curves.

Based on published evidence that as many as 43% of r/r FL patients were found to be progression free at 5 years following CAR-T therapy (26), a piecewise extrapolation was used for axi-cel curves to model a proportion of the population experiencing long-term survival. Exponential models for axi-cel OS and PFS were used up to 5 years; after 5 years, OS and PFS were calculated as a weighted average with 60% of the population following the base case ZUMA-5 survival extrapolations and 40% of the population following general population survival after applying a standardized mortality ratio (SMR) adjustment of 1.09 (**Figure 2**) (27).

**Figure 3:**
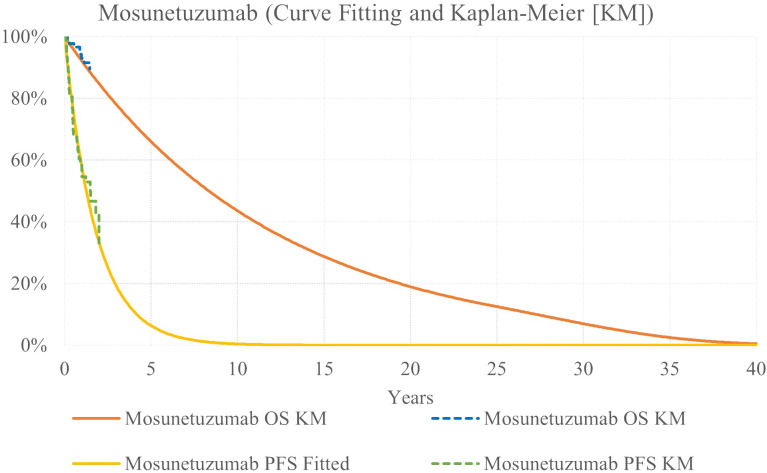
Mosunetuzumab OS and PFS KM Data and Parametric Survival Curves.

Mosunetuzumab survival was modeled via HR applied to axi-cel exponential survival curves (**Figure 3**). For PFS, the HR was estimated via MAIC to adjust for differences between the trial populations. The PFS HR was estimated to be 0.38 for axi-cel versus mosun (13). This HR was applied to axi-cel curves based on KM data to reflect the full ZUMA-5 trial population.

Because median OS was not reached for either treatment in available data (14, 25), a conservative assumption was implemented, reflecting an OS HR of 1.0. Because there is no evidence that bispecific monoclonal antibodies lead to long-term remission in r/r FL, base case analysis did not include a cure assumption at 5 years for the mosun arm.

### 2.3 Other model inputs

#### 2.3.1 Adverse events

Grade ≥3 adverse events (AE) reported from the ZUMA-5 and GO29781 trials that occurred in 5% or more patients were included in the model for costing purposes and to account for treatment-related disutilities.

It was assumed that all severe AEs related to axi-cel administration were treated during the initial inpatient admission per the ZUMA-5 trial protocol, except for hypogammaglobulinemia, which is a long-term AE and was thus incorporated with an additional cost. This approach prevents double counting AE management costs, as the initial inpatient cost is captured as part of the overall axi-cel treatment cost and would include the cost of AE management.

Grade ≥3 AE rates for mosunetuzumab treatment were retrieved from the pivotal clinical trial results (14). Because there were no initial inpatient admission, AE costs are applied separately for mosun. AE costs were calculated by multiplying the rate of each AE by the mean unit hospital commercial costs obtained from the US Department of Health & Human Services, HCUPnet - Healthcare Cost and Utilization project (28). Costs were inflated to 2023 USD based on inflation estimates from the US Bureau of Labor Statistics (BLS) (29).

#### 2.3.2 Health related quality of life

Time spent in each health state was quality-adjusted by multiplying with state-specific health utility values derived from the literature. A health state utility of 0.805, associated with complete response in iNHL patients (30, 31), was associated with the PF health state.

The PD health state utility differed for on-treatment and off-treatment, as patients who received additional treatment after failure of 3L therapy were assumed to experience worse quality of life compared to those who did not receive any treatment. For patients on treatment, the PD utility used in the model was 0.620 based on the combined health states of active disease, whereas the off-treatment utility was 0.736 based on relapsed FL (30).

#### 2.3.3 Costs

##### 2.3.3.1 Treatment costs in progression-free state

Treatment costs for axi-cel and mosun are summarized in **Table 1**. Axi-cel treatment costs consisted of axi-cel acquisition and hospitalization costs (which covers the costs of administration, monitoring, and treatment of all adverse events except hypogammaglobulinemia), conditioning chemotherapy, and leukapheresis.

**Table 1:** Treatment costs in progression-free state.

Cost	Value	Notes
Costs associated with axi-cel		
Leukapheresis	$1,468.00	Medicare unadjusted APC payment for CPT code 36511 (32)
Axi-cel acquisition cost	$462,000.00	Medispan PriceRx (33)
Conditioning chemotherapy	$1,436.49	Calculated value based on dosing regimen and schedule specified in Yescarta PI (34), drug prices from Medispan PriceRx (33), and administration unit costs from CMS fee schedules (35)
Axi-cel infusion - Administration (30 min IV)	$132.16	HCPCS 96413 from CMS Physician Fee Schedule (35)
Axi-cel infusion - Hospitalization LOS	13 days	Median LOS for initial hospitalization was 13 days (36)
Hospitalization unit cost (per day)	$3,918.45	HCUP Statistical Brief #125 from 2012 specifies a mean hospitalization cost per day due to NHL equal to $2,400, which is inflated to 2023 US dollars (37)
Costs associated with comparator arm		
Mosunetuzumab acquisition cost (30 mg/30 ml vial)	$17,821.78	Medispan PriceRx (33)

Mosun was given in 21-day cycles, with the first cycle consisting of step-up doses of 1 mg, 2 mg, and 60 mg, followed by a second cycle of 60 mg, and 30 mg cycles thereafter, to a recommended treatment duration of eight 30 mg cycles. The total cost of mosun was adjusted by a relative dose intensity of 98.7% (14). The total mosun drug cost was applied once for patients in the comparator arm in PFS at the first model cycle. The median OS for mosun was not reached in the median 18.3-month follow-up of the study, so applying costs per cycle does not reduce treatment costs substantially.

##### 2.3.3.2 Treatment costs in progressed state

Costs for subsequent lines of treatment are summarized in **Table 2**. FL patients initiated subsequent treatment after progressing from axi-cel or mosun treatments, respectively. Within the model, patients incurred a one-time treatment cost to account for the cost of all subsequent lines of therapy (LoT); each subsequent LoT is not explicitly modeled to retain analytic focus on the effect of primary interventions and to permit tractability of calculations given the assumption of nondifferential subsequent treatment series in each intervention arm.

**Table 2:** Treatment costs in progressed disease state.

Input	Value	Notes
Progressed disease treatment cost inputs		
Maximum number of subsequent LoT	7	SCHOLAR-5 Data on File (38)
Time between subsequent LoT (months)	10	
Median OS (months)	48	
Percentage of patients undergoing each subsequent LoT		
1^st^ subsequent LoT	44.4%	SCHOLAR-5 Data on File (38)
2^nd^ subsequent LoT	15.6%	
3^rd^ subsequent LoT	11.1%	
4^th^ subsequent LoT	11.1%	
Treatment cost at time to first progression	$170,930	Calculated
Other costs		
Health state costs (per month) Progression-free PD	$287 $443	Weighted monthly cost of inpatient visits, ED visits, and physician office visits (35)
Monitoring costs (per month)	$79	Weighted monthly cost of oncologist visit, complete blood count, and CT scan (35, 39)
End of life cost	$1,647	Calculated

To calculate the one-time PD treatment cost, a weighted average cost per course was calculated based on a market basket of available FL chemotherapy and hematopoietic stem cell transplant regimens weighted by the market shares for each treatment. This weighted average cost was multiplied by the proportion of patients transitioning to each subsequent LoT, and the resulting cost was applied once when patients entered PD. The proportion of patients transitioning to each subsequent LoT was based on treatment pattern data of patients in the SCHOLAR-5 study (38). SCHOLAR-5 is an international cohort from which data were extracted for r/r FL patients from 7 institutions in 5 countries who initiated a third or higher (3L+) line of therapy (LOT) after July 2014, and are reported in **Table 2**.

##### 2.3.3.3 Health state costs

**Table 2** summarizes the costs for additional health state costs. Other health state costs were considered for each arm, including inpatient visits, ED visits and physician office visits that may be incurred (35). The rate of monitoring during treatment was the same for both axi-cel and mosun, consisting of an oncologist visit, complete blood count every three months and CT scan every five months (40, 41).

##### 2.3.3.4 End of life costs

End of life costs were included in the model based on published literature. The costing approach involved using the median length of stay (LOS) in hospice, the daily cost of palliative care, and the percentage of patients using hospice. The median LOS in hospice was 12 days (42).

Daily cost of palliative care was calculated based on the 6 last months of life costs reported by Chastek and colleagues for US patients with lymphoma, inflated to 2023 US dollars (43).

### 2.4 Sensitivity analyses

A one-way sensitivity analysis was conducted in which key model parameters were varied by ± 20% of their base case values or using reported standard errors or confidence intervals if available, to test their impact on overall outcomes [incremental cost effectiveness ratio (ICER) and incremental costs].

A probabilistic sensitivity analysis was also generated by running 5,000 iterations of the model, with parameter values selected from distributions around default values. All parameters were assigned a default distribution, including normal, beta, log-normal, or gamma distributions depending on the type of data, and distributions reflected known standard errors where possible. An assumption of 10% variation was used directly to capture uncertainty around the default value where not reported.

### 2.5 Scenario analysis

Additional targeted scenarios analyses were explored to understand the impact of the underlying data. In one scenario, the mosun HRs were applied to the weighted (adjusted) ZUMA-5 24-month data to most exactly reflect the MAIC. In a second scenario, the mosun HRs were applied to extrapolated survival curves from longer-term ZUMA-5 data that has recently been published (48-month follow-up) (12, 15).

## 3 Results

### 3.1 Base case

The base case analysis estimated a 1.82 LY increase and a 1.89 QALY increase when comparing axi-cel to mosun in 3L+ r/r FL patients (**Table 3**). Both LY and QALY gains of axi-cel were attributed to the additional time spent in the PF health state relative to mosun. It should be noted that the higher incremental QALYs relative to LYs is because the majority of LYs for mosun are in PD whereas the majority of LYs for axi-cel are in PF, where patients’ utility is higher.

**Table 3:** Differential Effectiveness (LYs and QALYs; discounted).

	Axi-cel	Mosun	Incremental results (axi-cel – mosun)
**Total LYs**	**9.34**	**7.52**	**1.82**
PF LYs	6.42	1.60	4.81
PD LYs	2.92	5.92	-3.00
**Total QALYs**	**7.10**	**5.21**	**1.89**
PF QALYs	5.16	1.29	3.87
PD QALYs	1.94	3.92	-1.99

LY, life years; PF, progression-free; PD, progressed disease; QALY, quality-adjusted life years.

Total LY and QALYs are sums calculated from PF LY/QALYs and PD LY/QALYs

Progression-free survival for axi-cel patients was 6.42 LYs compared to 1.60 LYs for mosun, which resulted in a PF state cost increase of $257,113 primarily driven by the one-time cost of axi-cel treatment (**Table 4**). Axi-cel was also associated with cost-offsets in PD (-$52,416), driven by reduced treatment costs due to patients spending less time with PD, and had lower costs for subsequent treatment lines when compared to the mosun arm. Total incremental costs for axi-cel were $204,377, resulting in an ICER of $108,307/QALY gained.

**Table 4:** Base Case Results (discounted).

	Axi-cel	Mosun	Incremental results (axi-cel – mosun)
**Total costs**	***$655,088***	***$450,711***	***$204,377***
***Total PFS costs***	***$538,080***	***$280,967***	***$257,113***
Treatment	$515,972	$265,118	$250,854
Administration	$132	$2,579	-$2,447
Monitoring resources	$4,736	$1,525	$3,211
Adverse events*	$101	$6,226	-$6,126
Health state costs	$17,140	$5,519	$11,621
***Total PD costs***	***$116,048***	***$168,464***	***-$52,416***
Treatment	$101,721	$129,967	-$28,246
Administration	$1,090	$1,393	-$303
Monitoring resources	$2,006	$5,622	-$3,616
Health state costs	$11,231	$31,482	-$20,250
***End-of-life costs***	***$960***	***$1,280***	***-$320***
**Incremental cost-effectiveness ratio (ICER; Δ$/ΔQALY)**			**$108,307**

ICER, incremental cost-effectiveness ratio; PD, progressed disease; PFS, progression-free survival; QALY, quality-adjusted life years.

* The cost of managing adverse events that occur during administration of CAR-T are included in the inpatient admission costs.

Values in bold are summary values. Total PFS costs summarize all costs in the progression free state, Total PD costs summarize all costs associated with progressed disease, and end-of-life costs are associated with transition to death.

### 3.2 Sensitivity analyses

**Figure 4:**
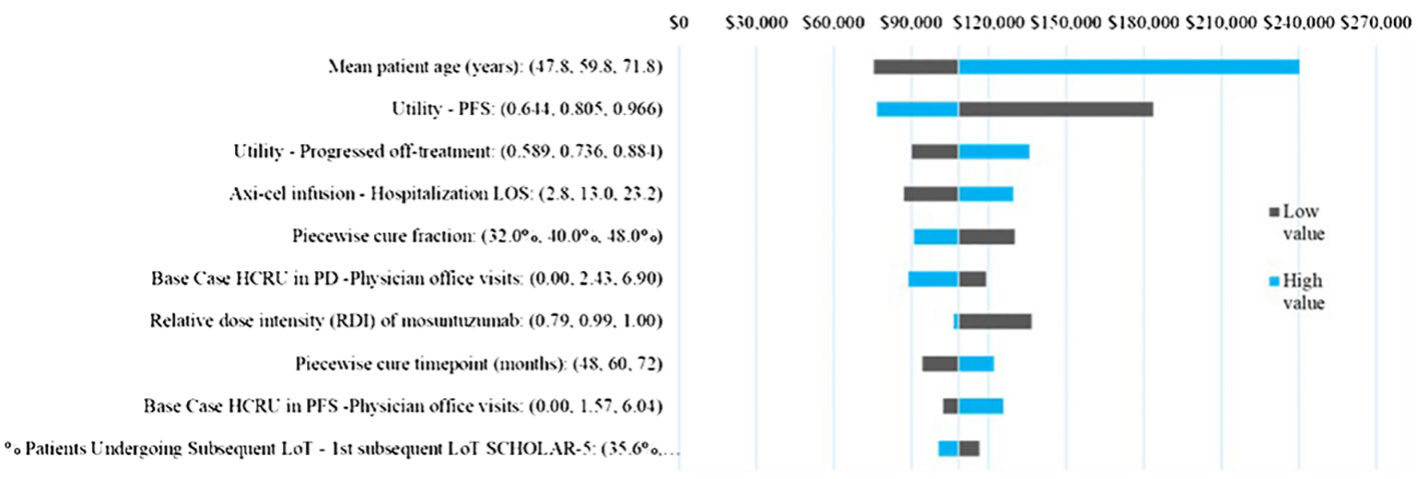
Tornado Diagram of OWSA Results.

Across all parameters varied in the one-way sensitivity analysis (**Figure 4**) the ICER varied between $240,255 and $75,624, with ICER ranges for all except for two parameters falling below $150,000/QALY. The ICER was most sensitive to mean patient age and the PF health utility, followed by the PD health state utility, PD health care resource use, axi-cel hospital LOS. The piecewise cure fraction, and relative dose intensity of mosun were also in the top 10 sensitive parameters but demonstrated limited impact on the ICER within the ±20% variation tested.

**Figure 5:**
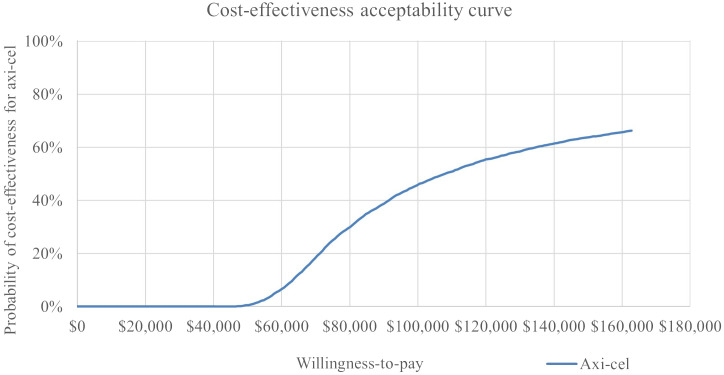
Cost-effectiveness Acceptability Curve from PSA Results.

In the probabilistic sensitivity analysis, axi-cel had an 64% probability of being cost-effective across 5,000 iterations using a $150,000 willingness-to-pay threshold. The cost effectiveness-acceptability curve is shown in **Figure 5**. A scatterplot of incremental costs versus incremental QALYs is shown in in the appendix.

### 3.3 Scenario analysis

In scenario 1, when using ZUMA-5 24-month data adjusted with weights as were used in the MAIC with mosun, the ICER also remained in the same range, at a value of $105,353 vs the base case of $108,307. The total LY and QALYs for axi-cel and mosun based on the weighted ZUMA-5 data were 10.26 and 8.74, and 7.83 and 5.98 respectively, for incremental LY and QALYs of 1.51 and 1.85. Total and incremental costs for axi-cel and mosun in this scenario were $650,429, $455,767, and $194,662, respectively.

In scenario 2, utilizing the HR values based on 24-month data with the most recent axi-cel follow-up data (48-month) resulted in similar ICER values. The total LY and QALYs for axi-cel and mosun based on 48-month follow-up data were 10.39 and 8.92, and 7.96 and 6.09, resulting in incremental LY and QALYs of 1.47 and 1.87. Total and incremental costs for axi-cel and mosun based on 48-month follow-up data were $648,285, $455,959, and $192,326, respectively. The ICER changed from $108,307 to $102,695 for analyses based on 24- and 48-month data, respectively.

**Figure 6:**
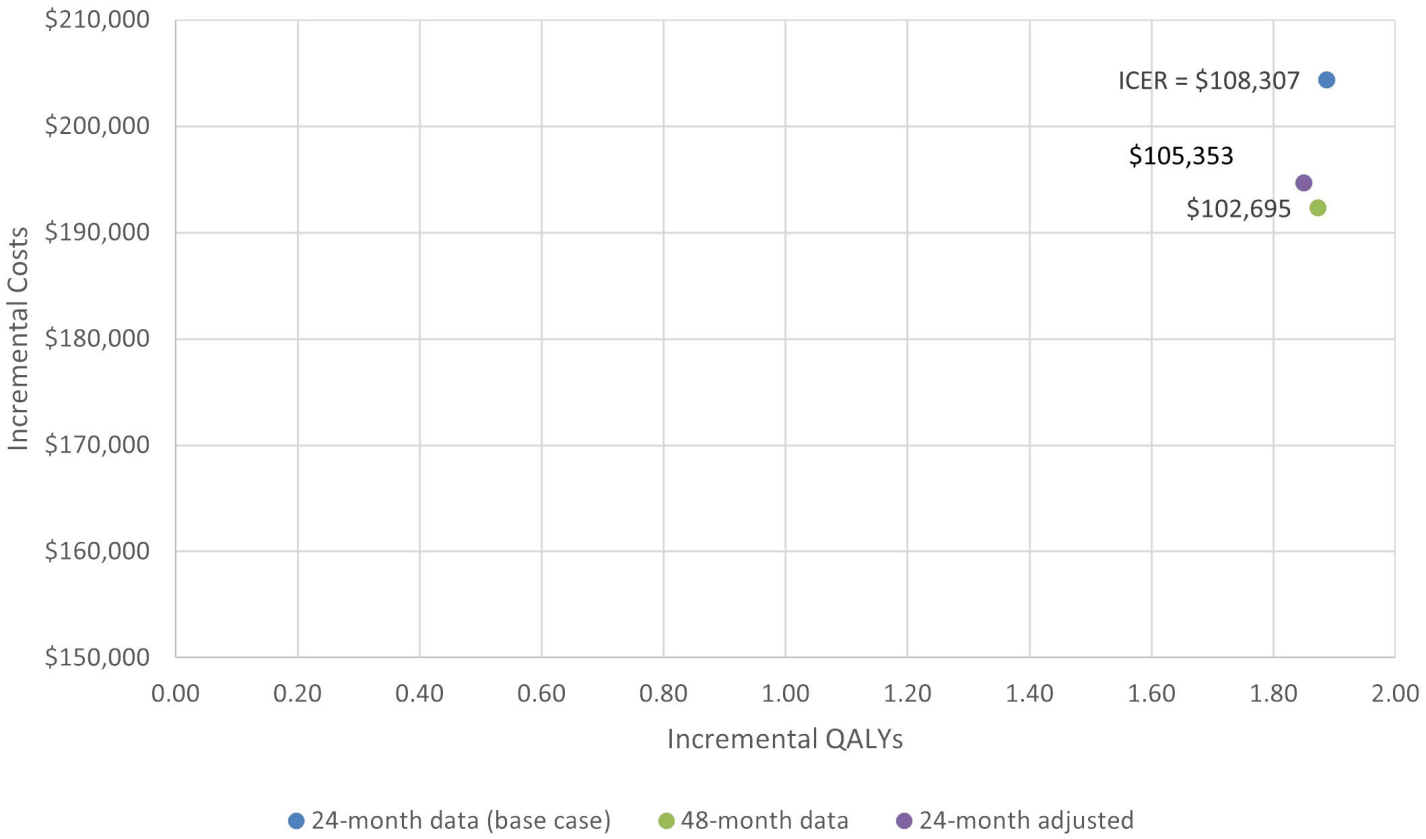
Cost-effectiveness plane for base case and scenario analysis.

The incremental costs, QALYs, and ICERS for the base case and two scenarios are shown in **Figure 6**.

## 4 Discussion

### 4.1 Summary

Results of this analysis indicate that axi-cel may be considered cost-effective at a willingness to pay threshold of $150,000/QALY when compared with mosun, with a base case deterministic ICER of $108,307/QALY, and 64% of analyses falling below the threshold when evaluated probabilistically. Mean patient age and quality of life utility values drove the largest changes in results when varied, while all other parameter ranges tested led to ICERs below $150,000/QALY. For patient age, the result indicates that axi-cel treatment has higher value when used early enough for patients to experience the benefits of longer survival. Similarly, altering utilities impacts the value of changing time spent in each state and thus intensifies estimated efficacy differences between treatments. Overall, axi-cel increases the time patients spend in PF state, thus improving their quality of life and offsetting some costs over a lifetime horizon. Given recently published 48-month ZUMA-5 follow-up data with median PFS of 57.3 months for the FL subgroup, compared to approximately 3.5 years in the 24-month data extrapolations, the current base case analysis could be considered a lower bound of possible treatment benefit due to axi-cel. The scenario based on the 48-month data demonstrates that the ICER would fall to $102,695/QALY.

These results contrast with the most recent publications on the cost-effectiveness of mosun for treatment of 3L+ r/r FL in the US. Both conference abstracts found mosun cost-effective or dominant compared to axi-cel, and dominant or cost-effective against other comparator treatments except for rituximab + lenalidomide (44, 45). Although our analysis had similar incremental treatment costs (incremental treatment costs of $257,113 vs $214,476 in Lin et al, and $284,453 in Matasar et al), the implementation of efficacy data differs. Although Matasar et al. used a similar 3-state partitioned survival model and a MAIC for clinical trial data, it did not report the matching method or the HRs and thus cannot be commented on in detail. In contrast, the MAIC cited in this study has been published in full detail (13). Moreover, the extrapolation of data in their analysis does not align with the assumptions in the current study. While the previous analysis assumed no cure effect for mosun, it likewise assumes no cure effect for CAR-T therapies, which fails to account for published evidence for treatments with this mechanism of action (26). That analysis therefore underestimates the potential benefits of CAR-T. Additionally, their analysis did not assume a treatment waning effect for mosun, which fails to align with the PFS differences and their own indirect treatment comparison (ITC) (45). For the study with Lin and colleagues, details are similarly lacking; it is unclear how or if the treatment efficacy was adjusted to account for different trial populations, nor was there any mention regarding assumptions for longer-term extrapolation. The study extrapolations do not align with long-term follow-up data generated for ZUMA-5; Lin estimated that less than 30% would be progression-free at 5 years, whereas the ZUMA-5 48-mo follow-up data used in scenario 2 shows that 53% are still progression-free and thus on a higher trajectory (15). Given the differences in extrapolation methods use in Lin compared to the current study, the value of axi-cel appears to be underestimated. The current analysis accounts for both the potential cure effect of CAR-T therapies and the shorter PFS of mosun, based on evidence from previous publications.

Alternatively, another recently published MAIC of mosun vs. axi-cel bolsters the findings of the current study, showing a PFS benefit for axi-cel that is consistent with our study findings (12). This study used mosun individual patient-level data from three trials, GADOLIN, CONTRALTO and NCT02257567, and found that mosun could be considered more favorable than tazemetostat in *EHZ2* wild-type patients for all outcomes. However, CAR-T therapies such as axi-cel would be favored for PFS, ORR and CR (44).

The current analysis is based on inpatient treatment of axi-cel patients, consistent with the ZUMA-5 trial, however, the results of this analysis needs to be considered in terms changing treatment patterns as outpatient administration of CAR-Ts and monoclonal antibodies becomes more common (11, 46). The pivotal trials for CAR-T were performed in the inpatient settings to anticipate adverse event management for cytokine release syndrome (CRS) and immune effector cell-associated neurotoxicity syndrome (ICANS). A recent evaluation of real-world treatment patterns show that approximately 19% of FL patients received CAR-T in the outpatient setting and among those who required a hospital admission within 30-days, the mean LOS was between 8.8 – 9.4 days, which is lower than the mean LOS of 14.8 days observed in ZUMA-5 (47, 48). The decision to administer CAR-T in the outpatient setting may depend on infusion time, expected time to CRS, and availability of outpatient monitoring (46, 49). Mosunetuzumab has been developed as an off-the-shelf outpatient therapy, and uses a step-up dosing regimen to mitigate CRS risk (11). As additional real-world evidence is obtained on costs and adverse events among patients with outpatient administered CAR-T therapy, re-evaluation of cost-effectiveness should be considered to quantify the impact of migrating CAR-T administration to the outpatient setting.

### 4.2 Limitations

As with any modeling study, limitations must be acknowledged. This study was based on clinical trial data with limited samples sizes, increasing uncertainty around model inputs. Additionally, the use of clinical trial data may impact generalizability to a real-world setting. However, the data are the only clinical evidence available and thus the best option to guide understanding of the value of these novel therapies.

In addition, due to the lack of a head-to-head comparison of axi-cel and mosun, a MAIC was used to estimate differential efficacy for PFS and OS between axi-cel and mosun. Although this alleviates some of the inconsistencies between trials and is the only available option based on current data availability, it cannot replace a true head-to-head comparison. However, the studies had considerable overlap between study populations leading to a fairly large effective sample size (ESS) for the MAIC and indicating a higher likelihood of finding stable and robust estimates (50). Additionally, the recently released 3-year data for mosun found lower PFS than for patients in ZUMA-5 at the same follow-up timepoint (14). Furthermore, due to limited mosun follow-up data, it was not possible to accurately estimate a HR for mosun OS curves. However, this is addressed by assuming that there is no difference in the shape of OS curves between the treatments. The present study also omits a cure fraction associated with mosun treatment; this is due to limited data about treatments with mosun’s mechanism of action. Conversely, the cure fraction of 40% used as the default for axi-cel after 5 years may actually be quite conservative, as 53% of patients are still progression-free at 48-months in ZUMA-5.

Finally, this study captures the treatment, administration, and AE costs associated with further progression via a market basket approach. This was based on SCHOLAR-5 data, and therefore reflects the set of current treatments other than CAR-T or bispecifics and thus costs for this patient population, allowing the model to retain focus on the initial treatment comparison while accounting for the impact of delaying progression.

Despite these limitations, this study provides an estimate of comparative effectiveness and cost-effectiveness of axi-cel for treatment in r/r FL patients who are 3L+ therapy, and thus adds to the set of information available to support complex treatment decisions.

### 4.3 Conclusions

This study finds that axi-cel is cost-effective compared to mosun at the commonly cited $150,000/QALY US willingness-to-pay threshold, with robust results across a range of sensitivity analyses accounting for parameter uncertainty. Although continued long-term follow-up will be necessary to reduce uncertainty about the proportion of patients experiencing long-term remission, axi-cel is expected to be an efficient use of resources compared to mosun, and thus an economically favorable addition to 3L+ treatment options for r/r FL in the US.

## Data availability statement

The original contributions presented in the study are included in the article/**Supplementary Material**. Further inquiries can be directed to the corresponding author.

## Author contributions

OO: Investigation, Validation, Writing – review & editing. MR: Conceptualization, Data curation, Funding acquisition, Methodology, Project administration, Supervision, Validation, Writing – review & editing. RZ: Formal analysis, Investigation, Methodology, Validation, Writing – review & editing, Writing – original draft. CF: Conceptualization, Methodology, Project administration, Validation, Writing – original draft, Writing – review & editing. BD: Formal analysis, Methodology, Validation, Writing – review & editing. AP: Data curation, Validation, Writing – review & editing. SB: Conceptualization, Formal analysis, Project administration, Supervision, Validation, Writing – original draft, Writing – review & editing.
