# Supplementary material for: Cost-effectiveness of treating relapsed or refractory 3L+ follicular lymphoma with axicabtagene ciloleucel vs mosunetuzumab in the United States
Source: Front Immunol. 2024 May 24;15:1393939. doi: 10.3389/fimmu.2024.1393939 (PMC11157123; doi:10.3389/fimmu.2024.1393939)
Supplement: Supplementary file 1 [file DataSheet_1.docx]

Supplementary Material

**Supplementary Figure 1. Incremental QALY-cost scatterplot of probabilistic sensitivity analysis results**

**Supplementary Figure 2. Axi-cel OS and PFS KM Data and Parametric Survival Curves (Scenario 1: MAIC-adjusted 24-month data)**

**Supplementary Figure 3. Axi-cel OS and PFS KM Data and Parametric Survival Curves (Scenario 2: 48-month data)**

Supplementary Table 1. Scenario 1 (MAIC Adjusted 24-Month Data): Differential Effectiveness (LYs and QALYs; Discounted)

|  | **Axi-cel** | **Mosun** | **Incremental results** |
| --- | --- | --- | --- |
| **Total LYs** | ***8.74*** | ***10.26*** | ***1.51*** |
| PF LYs | 1.92 | 7.50 | 5.58 |
| PD LYs | 6.82 | 2.75 | -4.07 |
| **Total QALYs** | ***5.98*** | ***7.83*** | ***1.85*** |
| PF QALYs | 1.55 | 6.04 | 4.49 |
| PD QALYs | 4.44 | 1.79 | -2.65 |

LY: life years; PF: progression-free; PD: progressed disease; QALY: quality-adjusted life years

Supplementary Table 2. Scenario 1 (MAIC Adjusted 24-Month Data): Detailed Cost Results (discounted)

|  | **Axi-cel** | **Mosun** | **Incremental results** |
| --- | --- | --- | --- |
| **Total costs** | ***$455,767*** | ***$650,429*** | ***$194,662*** |
| ***Total PFS costs*** | ***$282,416*** | ***$541,384*** | ***$258,968*** |
| Treatment | $265,118 | $515,977 | $250,859 |
| Administration | $2,635 | $132 | -$2,503 |
| Monitoring resources | $1,826 | $5,450 | $3,624 |
| Adverse events | $6,226 | $101 | -$6,126 |
| Health state costs | $6,610 | $19,724 | $13,114 |
| ***Total PD costs*** | ***$172,131*** | ***$108,151*** | ***-$63,980*** |
| Treatment | $127,985 | $94,720 | -$33,265 |
| Administration | $1,371 | $1,015 | -$356 |
| Monitoring resources | $6,481 | $1,881 | -$4,600 |
| Health state costs | $36,293 | $10,535 | -$25,758 |
| ***End-of-life costs*** | ***$1,220*** | ***$894*** | ***-$326*** |
| **Incremental cost-effectiveness ratio (ICER; Δ$/ΔQALY)** | | | **$105,353** |

ICER: incremental cost-effectiveness ratio; PD: progressed disease; PFS: progression-free survival; QALY: quality-adjusted life years

Supplementary Table 3. Scenario 2 (48-Month Data): Differential Effectiveness (LYs and QALYs; discounted)

|  | **Axi-cel** | **Mosun** | **Incremental results** |
| --- | --- | --- | --- |
| **Total LYs** | ***8.92*** | ***10.39*** | ***1.47*** |
| PF LYs | 2.04 | 7.84 | 5.81 |
| PD LYs | 6.88 | 2.55 | -4.33 |
| **Total QALYs** | ***6.09*** | ***7.96*** | ***1.87*** |
| PF QALYs | 1.64 | 6.31 | 4.67 |
| PD QALYs | 4.45 | 1.65 | -2.80 |

LY: life years; PF: progression-free; PD: progressed disease; QALY: quality-adjusted life years

Supplementary Table 4. Scenario 2 (48-Month Data): Detailed Cost Results (discounted)

|  | **Axi-cel** | **Mosun** | **Incremental results** |
| --- | --- | --- | --- |
| **Total costs** | ***$455,959*** | ***$648,285*** | ***$192,326*** |
| ***Total PFS costs*** | ***$282,940*** | ***$542,417*** | ***$259,477*** |
| Treatment | $265,118 | $515,977 | $250,859 |
| Administration | $2,652 | $132 | -$2,520 |
| Monitoring resources | $1,936 | $5,674 | $3,737 |
| Adverse events | $6,226 | $101 | -$6,126 |
| Health state costs | $7,007 | $20,534 | $13,527 |
| ***Total PD costs*** | ***$171,808*** | ***$104,983*** | ***-$66,825*** |
| Treatment | $127,267 | $92,480 | -$34,787 |
| Administration | $1,363 | $991 | -$373 |
| Monitoring resources | $6,542 | $1,744 | -$4,798 |
| Health state costs | $36,635 | $9,768 | -$26,867 |
| ***End-of-life costs*** | ***$1,211*** | ***$884*** | ***-$327*** |
| **Incremental cost-effectiveness ratio (ICER; Δ$/ΔQALY)** | | | **$102,695** |

ICER: incremental cost-effectiveness ratio; PD: progressed disease; PFS: progression-free survival; QALY: quality-adjusted life years
